# Multiple myeloma of young adult presented with paraplegia, rare case report in Saudi Arabia

**DOI:** 10.1259/bjrcr.20190008

**Published:** 2019-04-29

**Authors:** Ghaith Barefah, Nizar Adnan Almaghrabi, Rayan Elsini

**Affiliations:** 1 Body and musculoskeletal radiology consultant, King Fahad General Hospital, Jeddah, Saudi Arabia; 2 Umm AlQura University, Makkah, Saudi Arabia; 3 Al-Imam Muhammad Ibn Saud Islamic University, Riyadh, Saudi Arabia

## Abstract

Multiple myeloma is the second most common hematologic malignancy. It is characterized by the neoplastic proliferation of plasma cells in the bone marrow, leading to excessive production of monoclonal immunoglobulin. The mean age at diagnosis is 65 years. There are only a few cases of Multiple Myeloma arising in young population reported in the literature. We present a case of 33-years-old male presented with complete bilateral lower limbs paralysis and loss of sensation which were gradual in onset and accompanied by upper and lower back pain for 1 month. MRI of the whole spine show multiple infiltrative bone marrow high signal in T2 and STIR sequences involve C4 and the upper dorsal vertebral bodies and the spinous process of D4 with left para-spinal and large posterior epidural mass compress the spinal cord. CT guidance obtains three samples from the mass and placed in formalin in separate containers. Histopathology examination revealed neoplastic growth composed of Sheet of diffuse atypical plasma cells infiltrating fibro collagenous and adipose tissue. Although Multiple myeloma is a disease of elderly; it still could present in young age group. Histopathology examination is the gold standard for diagnosis.

## Introduction

Multiple myeloma is a condition characterized by the neoplastic proliferation of plasma cells in the bone marrow, leading to the excessive production of monoclonal immunoglobulin.^1^ Multiple myeloma accounts for 1% of all cancers and is the second most common hematologic malignancy after lymphoma.^2,3^ The incidence varies from 2 to 15/100.000, and it is more common in African origin population, The mean age at diagnosis is 65 years.^4^ Prevalence of Multiple myeloma in patients younger than 40 and 50 years old is 2 and 10%, respectively.^1,5^ Multiple myeloma is a well-known disease of elderly, yet it still should be considered in the differential diagnosis of young adults age group. The main symptoms and complications are Hypercalcemia, renal failure, fatigue, lytic lesion on imaging modalities and bone pain, elevated total serum protein concentration and/or presence of monoclonal protein in urine or serum, and symptoms of malignancy (*i.e.* weight loss, night sweats, anaemia).^1^ The objective of this case report is to describe the radiological presenting features of multiple myeloma in young adult patients versus lymphoma.

## Case presentation

33 years old male presented to emergency Department with complete bilateral lower limbs paralysis and loss of sensation which were gradual in onset and accompanied by upper and lower back pain for 1 month. The patient denied any history of trauma, headache, fever, blurred vision, night sweating, weight loss, loss of appetite, urine\stool incontinence, abdominal distension, constipation, and diarrhea. He had no previous surgeries, not on medications and has no history of allergy. There is no family history of the same presentation. He works in a carton factory. The patient demonstrated normal vital signs. There was tenderness on thoracic and lumbar spine. Neurological examination of the upper limbs was unremarkable while in lower limbs there was complete paralysis with loss of sensation bilaterally.

Laboratory examinations were remarkable for ESR of 120 and total protein of 9.90. Other tests were unremarkable including Complete blood count, Coagulation profile, C-reactive protein, Serum electrolytes, Liver functions tests, Kidney functions tests, Thyroid functions tests, Urinalysis, and Blood cultures. Serum protein electrophoresis showed mild hypoalbuminemia and marked monoclonal hypergammaglobulinemia. Immunofixation showed monoclonal gammopathy IgA λ type.

**Figure 1.**
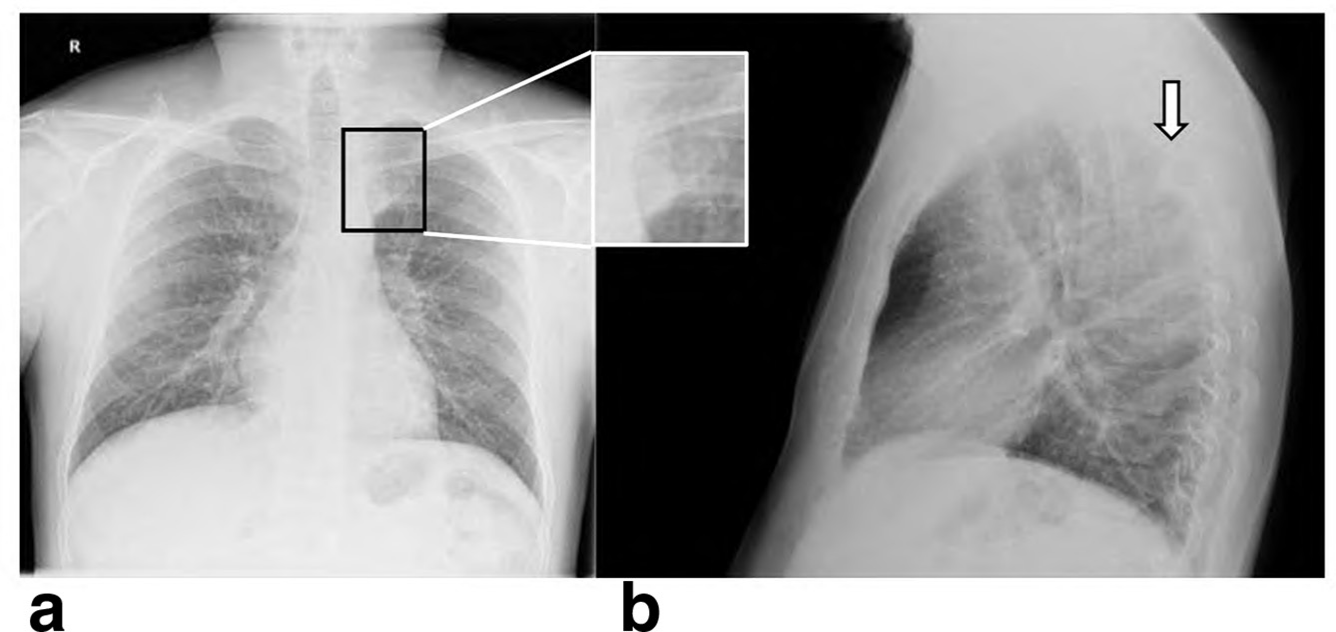
PA and Lateral, show left paraspinal posterior mediastinal opacity.

**Figure 2.**
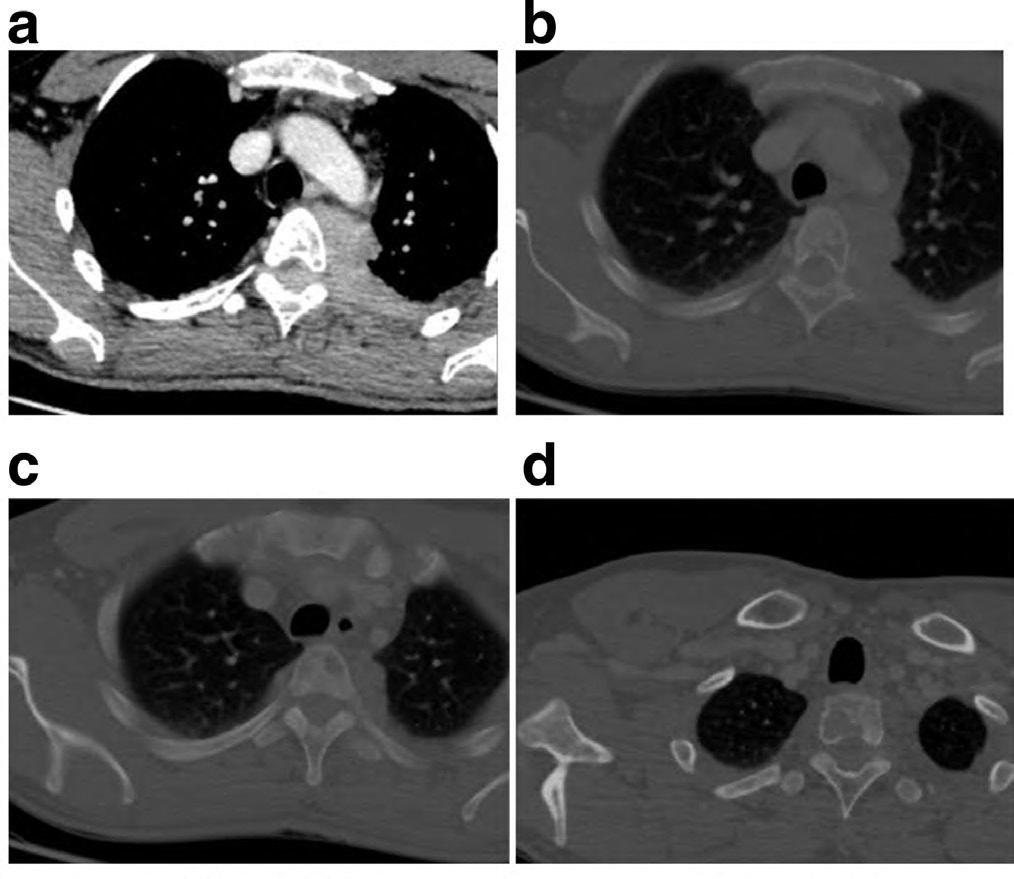
CT chest with contrast soft tissue window show left paraspinal mass.

**Figure 3.**
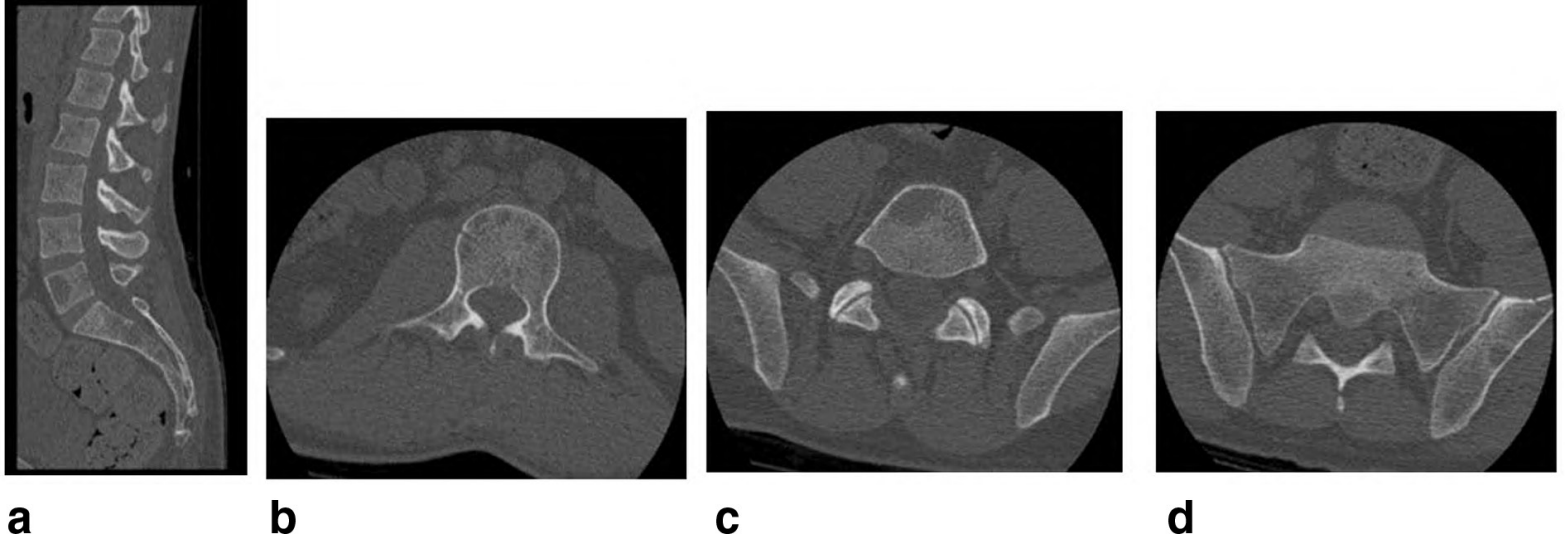
Sagittal reformat and axial CT scan of the cervical spine showed multiple lytic lesions.

**Figure 4.**
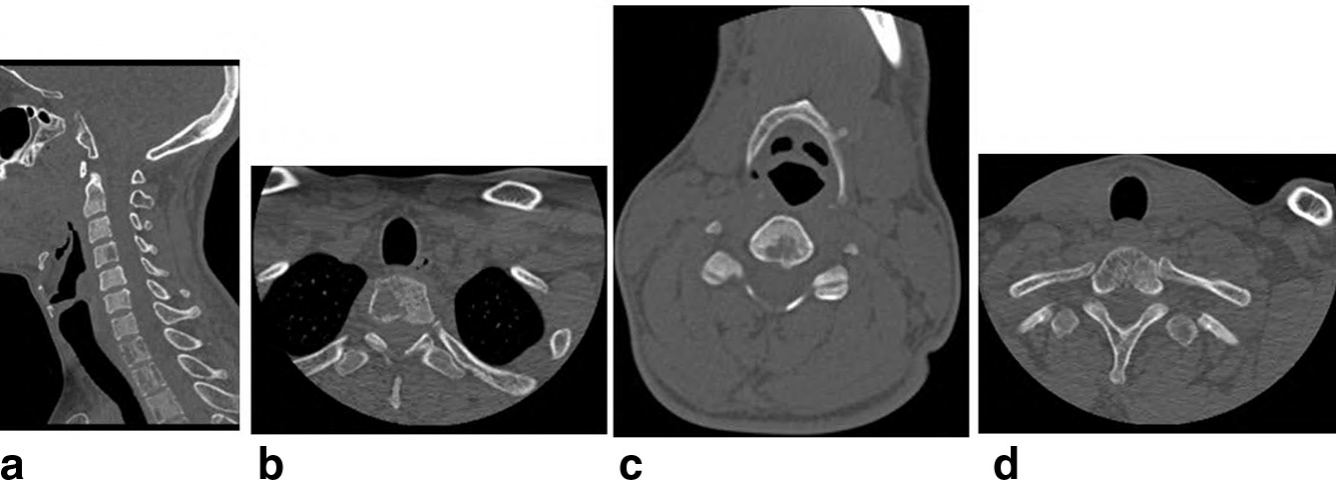
Sagittal reformat and axial CT scan of the cervical spine showed micronodular pattern and osteopenia with mini brain sign.

**X-ray chest** PA (Figure 1a) and Lateral (Figure 1b), show left paraspinal posterior mediastinal opacity above the aortic arch. CT chest with contrast (Figure 2a) soft tissue window show left paraspinal mass extend to the posterior epidural space through the left T2-T3 neural foramen, 2b, 2c and 2d bone window vertebral bodies punched out well defined multiple lytic lesions with endosteal scalloping. **CT scan for lumbar spine** was done and shows multiple lytic lesions involving the lumbar vertebral bodies, sacrum and left iliac bones. Largest lesion is seen in body of L5 which measures 1.5 × 1.5×2.2 cm in anteroposterior, transverse and craniocaudal diameters respectively. (Figure 3a Sagittal reformat, b, c and d axial). CT scan of the cervical spine showed multiple lytic lesions with micronodular pattern (Figure 4a, b, d) and osteopenia with mini brain sign (4c). **MRI** of the whole spine show multiple infiltrative bone marrow high signal in T2 and STIR sequences involve C4 and the upper dorsal vertebral bodies and the spinous process of D4 with left paraspinal and large posterior epidural mass compress the spinal cord, The left paraspinal mass show lobulated outline likely coming from the D4 vertebral body and pedicle measures 6.1 × 2.7×5.2 cm

**Figure 5.**
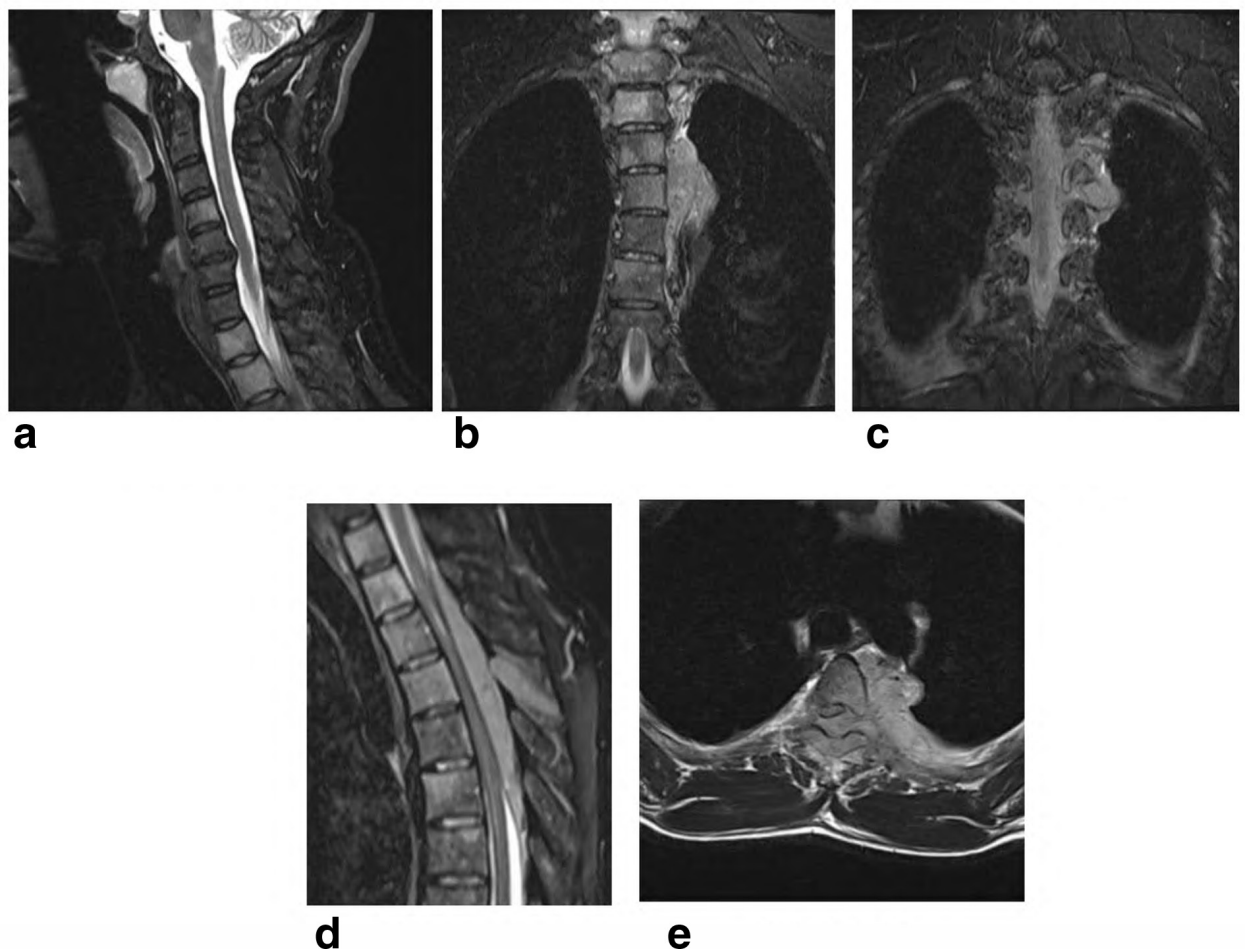
MRI of the whole spine show multiple infiltrative bone marrow with left paraspinal and large posterior epidural mass compress the spinal cord.

**Figure 6.**
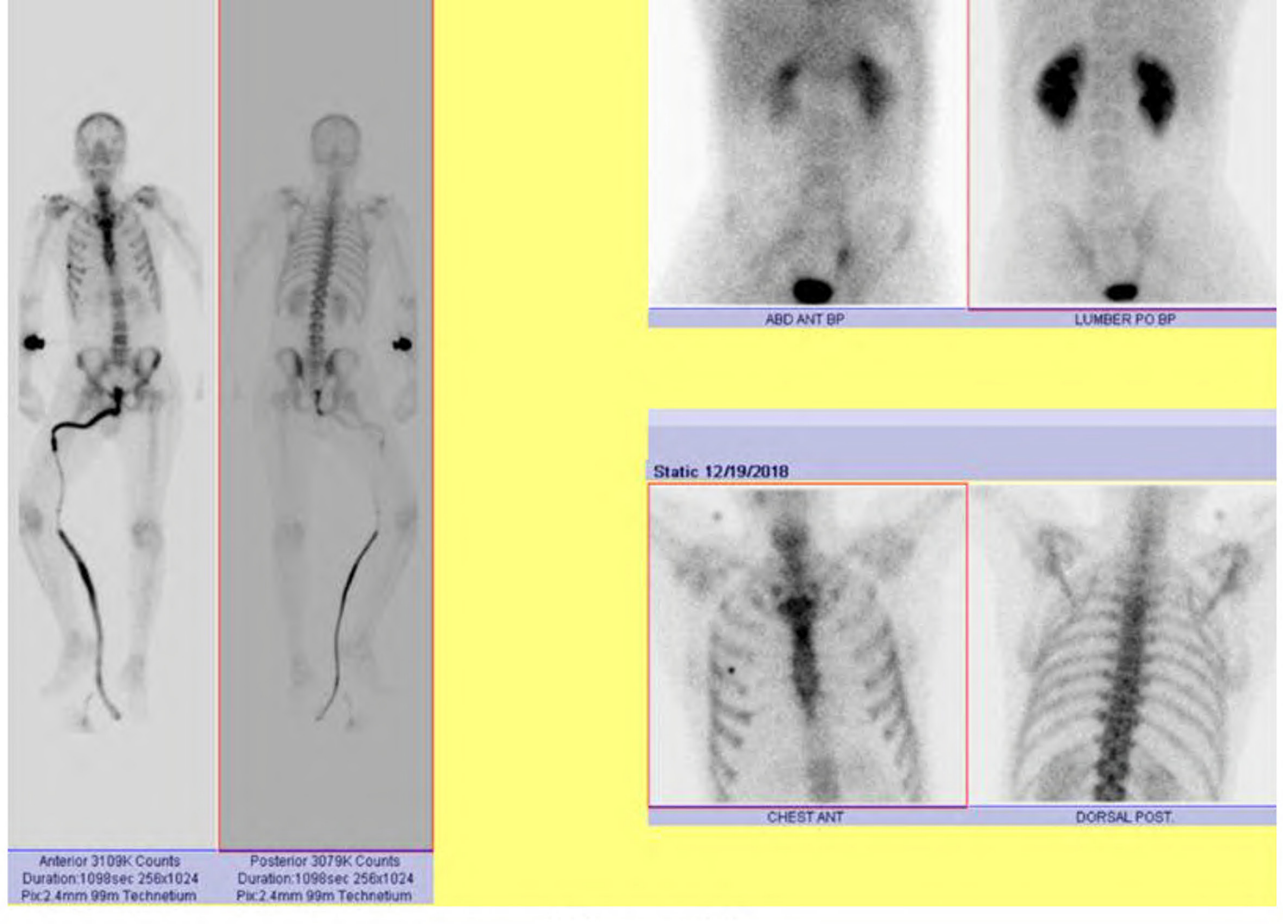
Delayed whole body bone scan.

**Figure 7.**
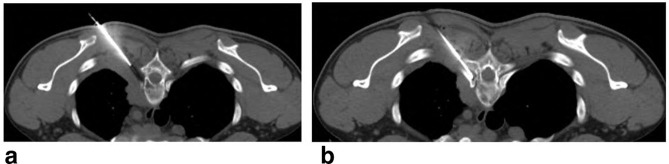
CT guidance biopsy left paraspinal mass.

The posterior epidural mass measures 1.7 × 1×11.2 from D2 to D6 likely coming from the spinous process and the left lamina of D4 (Figure 5a,b,c,d,e). **Delayed whole body bone scan** is performed and was unremarkable (Figure 6). Scanning of the chest shows left paraspinal mass Extending to the neural foramen between the second and third ribs. 15-gauge coaxial needle inserted under **CT guidance** and local anesthesia. A 16-gauge core biopsy needle was used to obtain three samples from the mass and placed in formalin in separate containers (Figure 7a,b).

**Figure 8.**
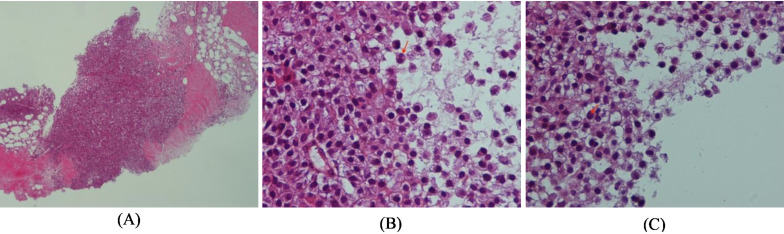
Histopathologyexamination revealed neoplastic growth.

Histopathology examination revealed neoplastic growth composed of Sheet of diffuse atypical plasma cells infiltrating fibro collagenous and adipose tissue ( Figure 8A). These atypical cells show variable degree of maturation from plasmablastic cells to plasma cells. Nuclei are enlarged showing both bi and multi neuclated cells with prominent nucleoli (Figure 8B). Perineuclear of and Russell bodies are also seen (Figure 8C).

Recently, the patient was diagnosed with renal failure; unfortunately, he was referred to tertiary center due to the lack of resources needed for further appropriate management.

## Discussion

Multiple myeloma is a disease of elderly. Prevalence of Multiple myeloma in patients younger than 40 years old is 2%.^1^ The term “*plasmacytoma*” means solitary plasma cell tumor without evidence of systemic spread, which has to be excluded by serum, bone marrow samples and imaging studies. Also when it arises outside the bone then it is called “extra osseous soft tissue myeloma”. While multiple myeloma is a disease which usually originates in the bone marrow, and spreads into the peripheral blood (plasma cell leukemia) or soft tissue.

*Monoclonal gammopathy of unclear significance* (*MGUS*) has to be distinguished from overt multiple myeloma. Its criteria includes a serum M-protein level less than 30 g l^−1^, clonal plasma cells in bone marrow less than 10% accompanied by a result of trephine biopsy that shows low levels of plasma cell infiltration [a low level of plasma cell infiltration in a trephine biopsy] (if present), no sign of any other B-cell proliferative disorders and no related tissue or organ damage, such as bone lesion or renal impairment.^6^

Multiple myeloma causes many complications; such as destruction of bones, typically in the spine, and fractures with minimal trauma “pathological fractures”. Fractures usually happen in tubular bones, mainly on cortical, not cancellous bone. Erode the cortex from inside outwards. So, the radiologist is required to anticipate impending fractures to assist the treating physician to take the best action required based on radiological images. For example, the presence of focal destructions is an important finding for follow-up and staging. The standard screening of the skeleton for bone destruction and osteoporosis is X-ray. Whole-body CT is superior to X-ray films in terms of finding focal bone destructions,^7^ but MRI is more sensitive^8^ and helps in evaluation of early infiltration of axial bone marrow and for following-up small lesions in 3- to 6-month period.^9^

In the past two decades, recommendations for the initial diagnostic imaging for evaluation have been modified as a result of research. Recently published guidelines recommend whole-body MR imaging for patients who have solitary plasmacytoma and a normal skeletal survey or MGUS.^10^ In addition, MR imaging is recommended for the evaluation of any patient with multiple myeloma and neurologic dysfunction, which may be indicative of epidural disease compressing the spinal cord.^11^

Usually, Multiple myeloma is characterized by osteolytic focal or diffuse lesions in radiographs in 90% of patients.^12–14^ It can involve any bone in the body including skull, pelvis, spine, ribs, and proximal long bones. If long bones are involved, endosteal cortical scalloping might be noticed which is an erosive cortex that is medullary in origin.^15^ There are other findings could present such as; well-defined, lytic, small, punched-out lesions found in calvaria and it might be associated with extra osseous soft tissue component..^10,13,16,17^

The radiological picture of Multiple myeloma can be similar to lymphoma or Metastases lesions and they are far more common than multiple myeloma, therefore, they might be difficult to discriminate.^18^ Osteolytic lesions are unlikely to be myeloma, especially in younger age group. To tell whether Osteolytic lesions in a given patient is plasmacytoma rather than lymphoma or Metastases can be very difficult using X-ray films or CT images, so CTG biopsy can be very helpful. So radiologically the differential diagnoses were Lymphoma, Metastatic lesion and Multiple myeloma. CTG biopsy was done to confirm the diagnosis of our patient by histopathology examination.

Increased uptake in bone scan might suggest lymphoma or metastasis rather than multiple myeloma which was negative in our patient along with clinical presentation including laboratory results which favors diagnosis of multiple myeloma.^10,19,20^ MRI helped to define and to differentiate patients with intermediate risk from those with a high risk for disease progression.^21^ Since the importance of MRI for sensitive detection and the prognostic significance of bone marrow infiltrates was illustrated, new myeloma management guidelines have been published Concerning diagnostic imaging, MRI and FDG-PET

This modified Durie and Salmon staging system (“Durie and Salmon Plus system; Table 1) is utilized by many clinicians since it permits better identification of early disease and helps to more precisely differentiate patients with stage II and III disease.^22,23^ Another alternative option for staging is the International Staging System, which does not utilize the use of any imaging criteria, therefore can be used in areas where imaging isn't available.^24^ Currently, there is no recommended protocol regarding post-treatment imaging of patients with multiple myeloma.^25^ Since lytic lesions do not heal, Skeletal survey has no role in monitoring the response to treatment. however, the skeletal survey may describe disease progression.^11^ Although post-treatment monitoring with CT, MR and FDG PET in combination or alone is not recommended currently, it may be useful in patients with focal symptoms, those enrolled in clinical trials, and those undergoing aggressive chemotherapy.^26,27^

**Table 1.** Durie and Salmon Plus System for Staging of Multiple Myeloma^22^ (Table 1)

**Stage**	**Laboratory findings**	**Imaging findings**
IA	≥10% plasma cells	Limited disease or plasmacytoma
IB	≥10% plasma cells, end organ damage	Mild diffuse disease,<5 focal lesions,
IIA, IIB	≥10% plasma cells, end organ damage	Moderate diffuse disease, 5–20 focal lesions
IIIA, IIIB	≥10% plasma cells, end organ damage	Severe diffuse disease,>20 focal lesions

The standard treatment in young patients with multiple myeloma Since the end of the 90 s is high dose chemotherapy with autologous stem-cell transplantation. The median age of survival is about five years.^28^ Among all age groups, the median duration of survival usually varies from 2 to 3 years. However, it seems that multiple myeloma in young patients is not associated with a worse survival or prognosis. The median survival duration among patients younger than 40 years was reported to be longer by 54 months.^29^ In another analysis done on 8860 patients with multiple myeloma aged 50 years of age and older compared with 1689 patients younger than 50 years, the survival was significantly longer in the younger patients (3.7 *vs* 5.2 years; *p* < .001).^30^

## Conclusion

We present a case of 33-year-old male with Multiple myeloma presented initially with complete lower limb paralysis with loss of sensation bilaterally. Pre-operative radiological diagnosis is usually difficult to make. Although Multiple Myeloma is rare to occur in this age group, it should be in the differential diagnoses of osteolytic lesions along with lymphoma and metastasis. We emphasize that biopsy is necessary in establishing the accurate diagnosis.

## Learning points

- Multiple myeloma is a disease of elderly; The mean age at diagnosis is 65 years.
- Multiple Myeloma is rare to occur in young age group, it should be in the differential diagnoses of osteolytic lesions along with lymphoma and metastasis.
- Increased uptake in bone scan might suggest lymphoma or metastasis rather than multiple myeloma.
- Recommend whole-body MR imaging for patients who have solitary plasmacytoma and a normal skeletal survey or MGUS
- Biopsy is necessary in establishing the accurate diagnosis.
